# Causal impact of gut microbiota and associated metabolites on pulmonary arterial hypertension: a bidirectional Mendelian randomization study

**DOI:** 10.1186/s12890-024-03008-7

**Published:** 2024-04-17

**Authors:** Xin Li, Jiang-Shan Tan, Jing Xu, Zhihui Zhao, Qing Zhao, Yi Zhang, Anqi Duan, Zhihua Huang, Sicheng Zhang, Luyang Gao, Yue Jin Yang, Tao Yang, Qi Jin, Qin Luo, Yanmin Yang, Zhihong Liu

**Affiliations:** 1 Center for Pulmonary Vascular Diseases, Fuwai Hospital, National Center for Cardiovascular Diseases, Chinese Academy of Medical Sciences and Peking Union Medical College, No.167 Beilishi Rd, Xicheng District, Beijing, 10003 China; 2 Emergency and Critical Care Center, Fuwai Hospital, National Center for Cardiovascular Diseases of China, Chinese Academy of Medical Sciences and Peking Union Medical College, Beijing, China; 3 State Key Laboratory of Cardiovascular Disease, Department of Cardiology, Fuwai Hospital, National Center for Cardiovascular Diseases, Chinese Academy of Medical Sciences and Peking Union Medical College, Beijing, China; 4 Department of Genetics, University Medical Center Groningen, University of Groningen, Groningen, The Netherlands; 5 Department of ICU, Sichuan Provincial People’s Hospital, University of Electronic Science and Technology of China, Chengdu, China; 6 University of Electronic Science and Technology of China, Chengdu, China; 7 Department of Cardiology, Zhongshan Hospital, Fudan University, Shanghai Institute of Cardiovascular Diseases, Shanghai, China

**Keywords:** Mendelian randomisation, Pulmonary arterial hypertension, Gut microbiota, Short-chain fatty acids, Trimethylamine N-oxide

## Abstract

**Background:**

Patients with pulmonary arterial hypertension (PAH) exhibit a distinct gut microbiota profile; however, the causal association between gut microbiota, associated metabolites, and PAH remains elusive. We aimed to investigate this causal association and to explore whether dietary patterns play a role in its regulation.

**Methods:**

Summary statistics of gut microbiota, associated metabolites, diet, and PAH were obtained from genome-wide association studies. The inverse variance weighted method was primarily used to measure the causal effect, with sensitivity analyses using the weighted median, weighted mode, simple mode, MR pleiotropy residual sum and outlier (MR-PRESSO), and MR-Egger methods**.** A reverse Mendelian randomisation analysis was also performed.

**Results:**

*Alistipes* (odds ratio [OR] = 2.269, 95% confidence interval [CI] 1.100–4.679, *P* = 0.027) and *Victivallis* (OR = 1.558, 95% CI 1.019–2.380, *P* = 0.040) were associated with an increased risk of PAH, while *Coprobacter* (OR = 0.585, 95% CI 0.358–0.956, *P* = 0.032), *Erysipelotrichaceae (UCG003)* (OR = 0.494, 95% CI 0.245–0.996, *P* = 0.049), *Lachnospiraceae (UCG008)* (OR = 0.596, 95% CI 0.367–0.968, *P* = 0.036), and *Ruminococcaceae (UCG005)* (OR = 0.472, 95% CI 0.231–0.962, *P* = 0.039) protected against PAH. No associations were observed between PAH and gut microbiota-derived metabolites (trimethylamine N-oxide [TMAO] and its precursors betaine, carnitine, and choline), short-chain fatty acids (SCFAs), or diet. Although inverse variance-weighted analysis demonstrated that elevated choline levels were correlated with an increased risk of PAH, the results were not consistent with the sensitivity analysis. Therefore, the association was considered insignificant. Reverse Mendelian randomisation analysis demonstrated that PAH had no causal impact on gut microbiota-derived metabolites but could contribute to increased the levels of *Butyricicoccus* and *Holdemania*, while decreasing the levels of *Clostridium innocuum, Defluviitaleaceae UCG011, Eisenbergiella,* and *Ruminiclostridium 5*.

**Conclusions:**

Gut microbiota were discovered suggestive evidence of the impacts of genetically predicted abundancy of certain microbial genera on PAH. Results of our study point that the production of SCFAs or TMAO does not mediate this association, which remains to be explained mechanistically.

**Supplementary Information:**

The online version contains supplementary material available at 10.1186/s12890-024-03008-7.

## Research in context

### Evidence before this study

Previous studies have reported that patients with pulmonary arterial hypertension (PAH) exhibit a distinct gut microbiota profile, characterised by a reduction in the number of bacteria that produce short-chain fatty acids. Moreover, gut microbiota-derived metabolites, such as trimethylamine N-oxide, are elevated in patients with PAH and are associated with an unfavourable prognosis. However, the causal association between gut microbiota, associated metabolites, and PAH remains elusive.

### Added value of this study

In this study, we aimed to investigate the causal relationship between gut microbiota, associated metabolites, and PAH and explore whether dietary patterns mediate this causal relationship using bidirectional Mendelian randomisation. The gut microbiota exhibits a potential protective effect against PAH, including *Coprobacter, Erysipelotrichaceae (UCG003), Lachnospiraceae (UCG008),* and *Ruminococcaceae (UCG005)*. In contrast, *Alistipes* and *Victivallis* were associated with an increased risk of PAH. Furthermore, PAH also had suggestive effects on gut microbiota, including the elevation of *Butyricicoccus* and *Holdemania* and the reduction of *Clostridium innocuum, Defluviitaleaceae UCG011, Eisenbergiella,* and *Ruminiclostridium 5*. However, we did not observe a significant causal association between gut microbiota-dependent metabolites (trimethylamine N-oxide and its precursors), short-chain fatty acids, and PAH. Moreover, dietary patterns were not associated with PAH, suggesting that dietary patterns do not mediate the association between gut microbiota, associated metabolites, and PAH.

### Implications of the available evidence

Genetically predicted gut microbiota has a suggestive causal effect on PAH, and the underlying mechanism may be attributed to alternative factors rather than the production of short-chain fatty acids and trimethylamine N-oxide. Further studies are warranted to elucidate the mechanisms underlying the relationship between gut microbiota and PAH.

## Background

Pulmonary arterial hypertension (PAH) is characterised by the chronic elevation of pulmonary arterial pressure and remodelling of the pulmonary arteries, which can ultimately lead to heart failure and mortality [1]. Several factors contributing to the development of PAH have been identified, including genetic susceptibility, underlying cardiovascular diseases, toxic exposure, and inflammation [2]. Recent studies have revealed that gut microbiota and its associated metabolites play vital roles in the pathogenesis of PAH. The gut microbiota, together with its metabolites, participates in various metabolic processes, such as cholesterol accumulation, impaired glucose tolerance, and elevated inflammatory responses, all of which promote the progression of PAH [3, 4]. Previous studies reported that patients with PAH exhibit a distinct gut microbiota profile characterised by a reduction in bacteria that produce short-chain fatty acids (SCFAs), including *Coprococcus, Lachnospiraceae, Eubacterium,* and *Clostridia* [5]. Moreover, gut microbiota-derived metabolites, namely trimethylamine N-oxide (TMAO) and its precursors choline, betaine, and carnitine, are elevated in patients with PAH and are associated with an unfavourable prognosis [3–5].

Despite the growing body of research examining the association between gut microbiota, its derived metabolites, and PAH, the majority of these studies have been cross-sectional or observational in nature and were unable to establish a causal relationship between PAH and gut microbiota. Additionally, the observed association between gut microbiota and PAH in cross-sectional or observational studies might be affected by reverse causation bias or confounders, such as dietary patterns. Moreover, the small sample sizes of these studies limit the robustness and generalisability of the conclusions. Therefore, the causality between gut microbiota, associated metabolites, and PAH warrants further investigation.

Mendelian randomisation, which introduces genetic variants as instrumental variables, is widely used to identify the causal effects of exposure on particular outcomes. Compared to traditional observational or cross-sectional studies, Mendelian randomisation is less prone to confounding because genetic alleles are randomly allocated during gametogenesis. Furthermore, Mendelian randomisation can mitigate the potential for reverse causation bias, as genotypes are determined prior to disease onset [6]. Therefore, in this study, we aimed to use a two-sample Mendelian randomisation method to investigate the causal relationship between gut microbiota, associated metabolites, and PAH. We also explored whether dietary patterns mediated this causal relationship.

## Methods

### Study design

**Fig. 1:**
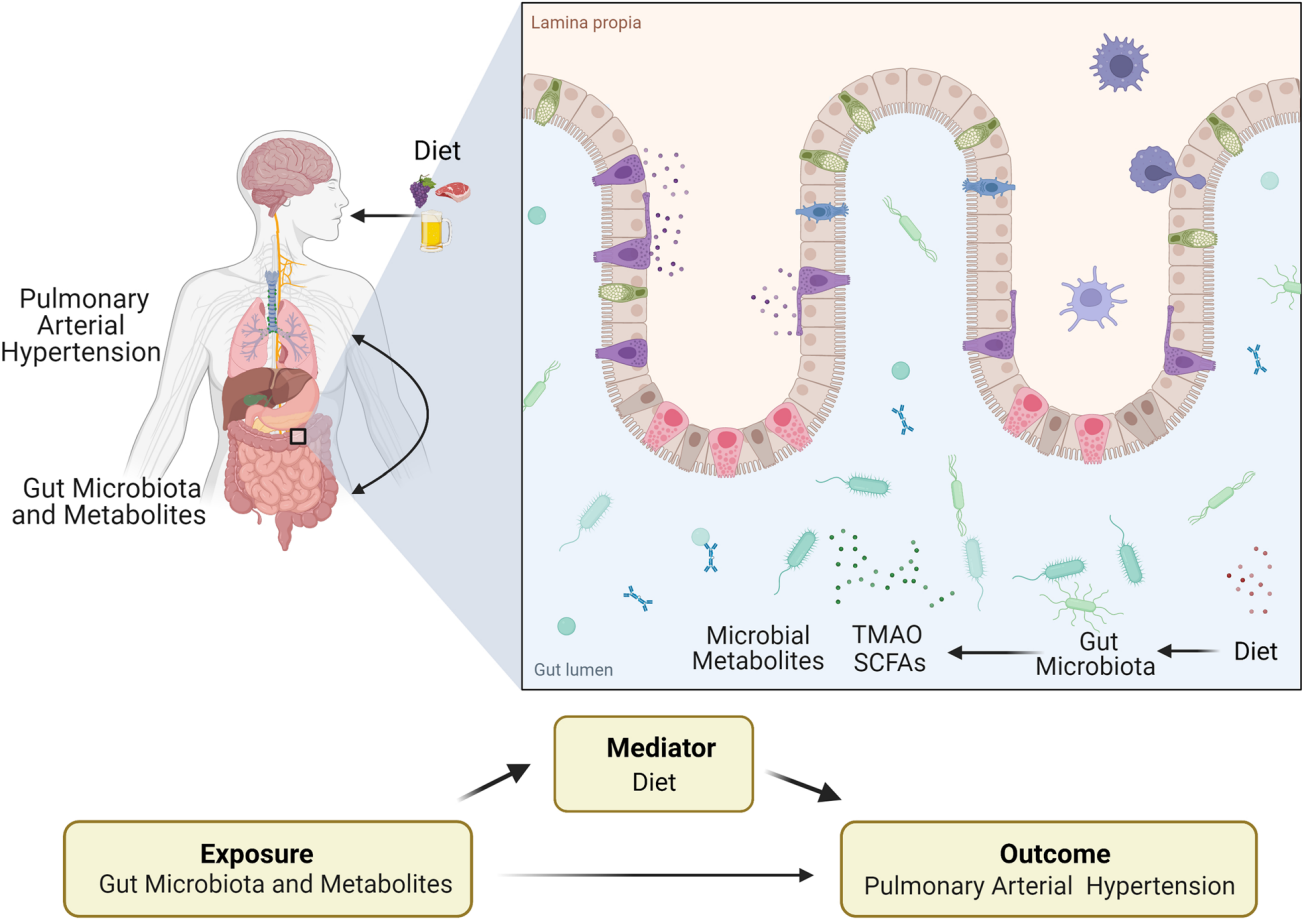
Study overview. Legend: SCFA, short chain fatty acids; TMAO, trimethylamine N-oxide. The figure illustrates the design of the study. In this study, we aim to investigate the causal relationship between gut microbiota, associated metabolites and PAH, and explore whether dietary patterns mediate the above causal relationship using two-sample Mendelian randomization

Single nucleotide polymorphisms (SNPs) used as instrumental variables should satisfy three assumptions: (i) the SNP correlates with exposure, namely gut microbiota, associated metabolites, and dietary patterns; (ii) the SNP does not correlate with confounding variables that could affect the association between exposure and PAH; and (iii) the SNP correlates with PAH exclusively through exposure rather than through other pathways. We explored the causal relationship between gut microbiota, associated metabolites, and PAH. We also evaluated whether dietary patterns mediated this association (Fig. 1).

### Data sources

We obtained the genetic variants for gut microbiota from the current largest genome-wide meta-analysis of gut microbiota composition, which was conducted by the MiBioGen consortium (https://mibiogen.gcc.rug.nl/) [7]. The study included 18,340 participants from 24 cohorts, most of whom were European [7]. The V1–V2, V3, and V4 variable regions of the 16S rRNA gene were used to identify microbial composition. The lowest taxonomic level in this study was at the genus level. This study encompassed 131 genera, each exhibiting an average abundance exceeding 1%. Of these, 12 genera were unknown. Consequently, this study included 119 genera.

To reduce the bias introduced by the study population, we limited the analytical studies to European populations. We searched for genetic variants of gut microbiota-derived metabolites, including TMAO, choline, betaine, carnitine, SCFAs, dietary patterns, and PAH, using genome-wide association studies (GWAS). The GWAS used to extract the summary-level data are detailed in Table 1 and Supplementary Table S1. This study was approved by ethic committee of Fuwai Hospital, Beijing, China.

**Table 1:** Causal effect of gut microbiota and dependent metabolite on pulmonary arterial hypertension

Exposure	Outcome	Method	Num of SNP	OR	95% CI	*P* value
Alistipes	PAH	IVW	51	2.269	1.100–4.679	0.027
Coprobacter	PAH	IVW	46	0.585	0.358–0.956	0.032
Erysipelotrichaceae (UCG003)	PAH	IVW	46	0.494	0.245–0.996	0.049
Lachnospiraceae (UCG008)	PAH	IVW	49	0.596	0.367–0.968	0.036
Ruminococcaceae (UCG005)	PAH	IVW	59	0.472	0.231–0.962	0.039
Victivallis	PAH	IVW	33	1.558	1.019–2.380	0.040
Choline	PAH	IVW	95	1.535	1.015–2.322	0.043
Carnitine	PAH	IVW	278	1.429	0.055–36.824	0.830
Betaine	PAH	IVW	59	4.678	0.676–32.376	0.118
TMAO	PAH	IVW	58	1.077	0.957–1.212	0.219
Acetate	PAH	IVW	139	0.808	0.272–2.401	0.701
Indolepropionate	PAH	IVW	47	1.385	0.276–6.937	0.692
3-Hydroxybutyrate	PAH	IVW	125	3.002	1.035–8.708	0.043

*SNP* single nucleotide polymorphism, *OR* odds ratio, *CI* confidence interval, *TMAO* trimethylamine N-oxide, *PAH* pulmonary arterial hypertension, *IVW* Inverse variance weighted. For the results of sensitivity analysis, please kindly refer to Supplementary Table S2 & Supplementary Table S6

### SNP selection

The SNPs for instrumental variables were selected as follows: (i) the SNP correlated with gut microbiota at locus-wide significance with *P* < 5 × 10^−5^ [8, 9]; (ii) to ensure the independence of instrumental variables, the selected SNPs were clumped using 1000 Genomes Project European sample data as a reference, employing a clumping window of 10,000 kb and a linkage disequilibrium R^2^ threshold of < 0.001; (iii) the instrument strength was evaluated using the F-statistic, and the variance was quantified using r^2^. SNPs with an F statistic of < 10 were excluded because of weak instrument bias [10].

### Data analysis

The inverse variance weighted (IVW) method was primarily used to measure the causal effect of gut microbiota, associated metabolites, and dietary patterns on PAH, which demonstrates a combined causal estimate from each SNP [11]. The weighted median, weighted mode, simple mode, MR-PRESSO, and MR-Egger analyses were performed to examine the validity of the results. A stable causal association can only be established when the sensitivity analysis yields results consistent with those of the IVW method [12]. The weighted median method can provide unbiased estimates of causal associations when half of the instrumental variables are invalid. The MR-PRESSO approach was applied to detect SNP outliers and to provide estimates after removing outliers [13]. Leave-one-out analysis was used to evaluate whether a variant drove the correlation between exposure and outcome by removing a single SNP each time [12, 14]. The MR-Egger regression was applied to detect pleiotropy, and an intercept that was significantly distinct from zero indicated the existence of pleiotropy [15]. The slope of the MR-Egger regression provided causal estimates after the pleiotropy correlation. The Cochrane Q statistic was used to evaluate heterogeneity [12]. Given the multiple tests used in this study, the Bonferroni-corrected threshold of statistical significance was *P* < 0.0004 (0.05/131). A *P* value between 0.0004 and 0.05 was regarded as a suggestive association. All statistical analyses were performed using the R software (version 4.0.5; R Foundation for Statistical Computing, Vienna, Austria). MR analyses were performed using TwosampleMR [16] and MR-PRESSO [13] packages.

## Results

**Fig. 2:**
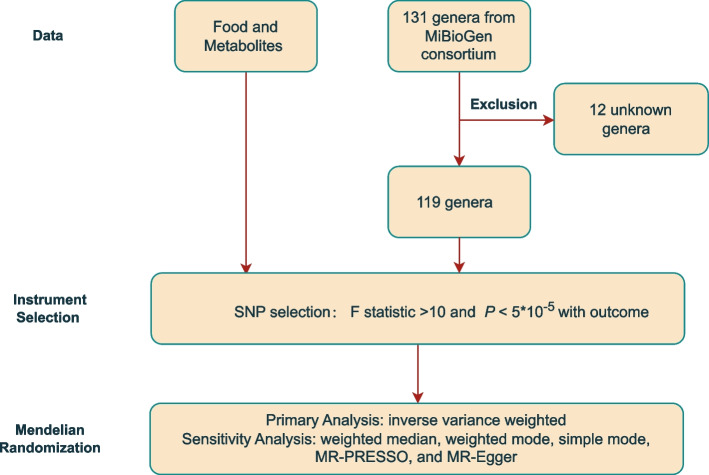
Flow Chart of the Study Design. Legend: SNP, single nucleotide polymorphisms

Figure 2 demonstrates the study design. The features of the selected SNPs in gut microbiota and its associated metabolites are shown in Supplementary Table S1. A total of 5394 SNPs were selected as instrumental variants for 119 bacterial genera; 311 SNPs for SCFAs, including acetate, indolepropionate, and 3-hydroxybutyrate; and 490 SNPs for TMAO, along with its precursors (Supplementary Table S2).

### Causal effect of gut microbiota on PAH

**Fig. 3:**
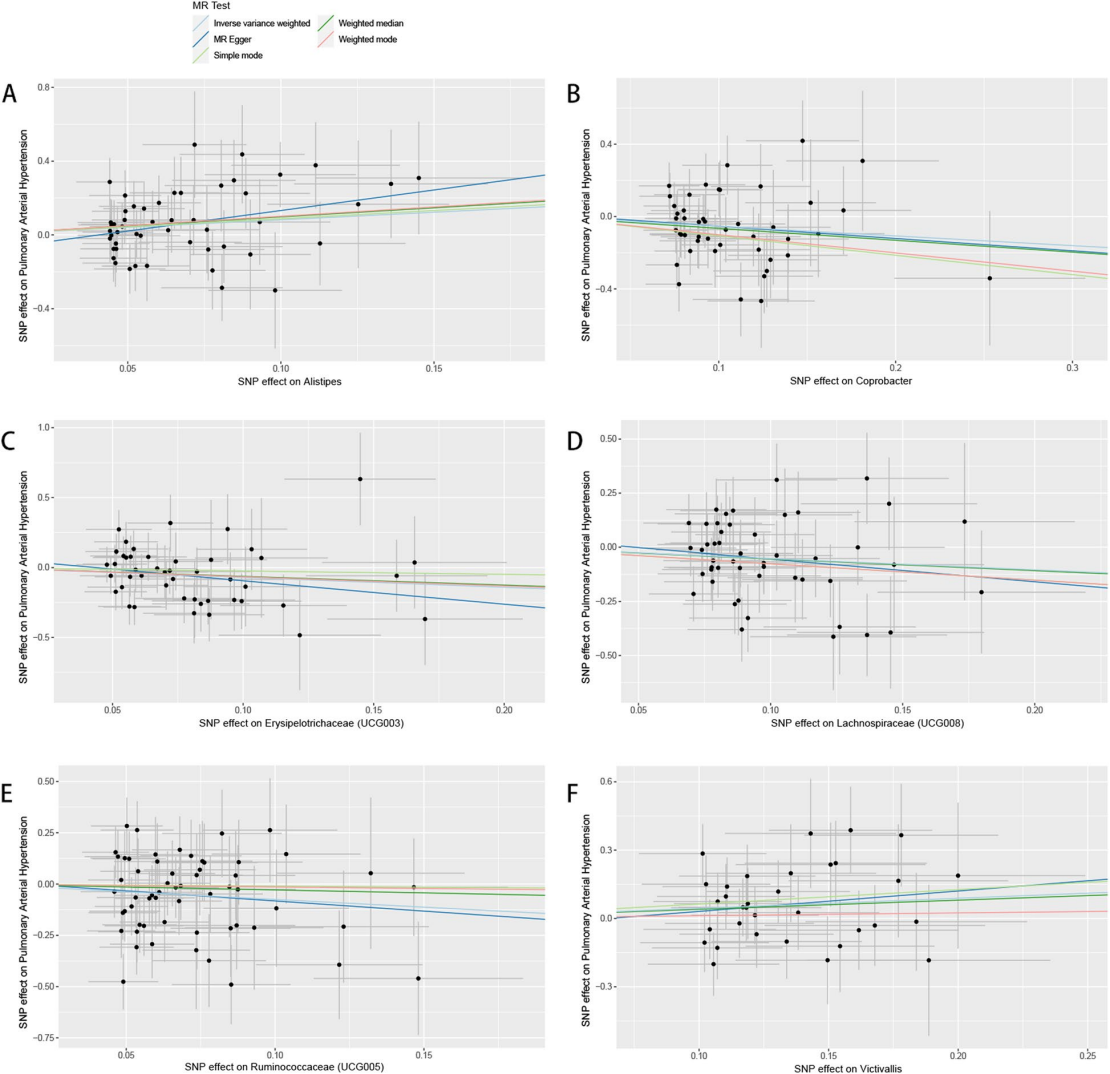
Scatter plot of MR results of bacteria on pulmonary arterial hypertension. Legend: Each dot represents the size of the SNP exposure impact in standard deviation units with the accompanying standard error. The lines represent the effect size calculated by the mendelian randomization method with the corresponding color. MR, Mendelian randomization; SNP, single nucleotide polymorphisms

**Fig. 4:**
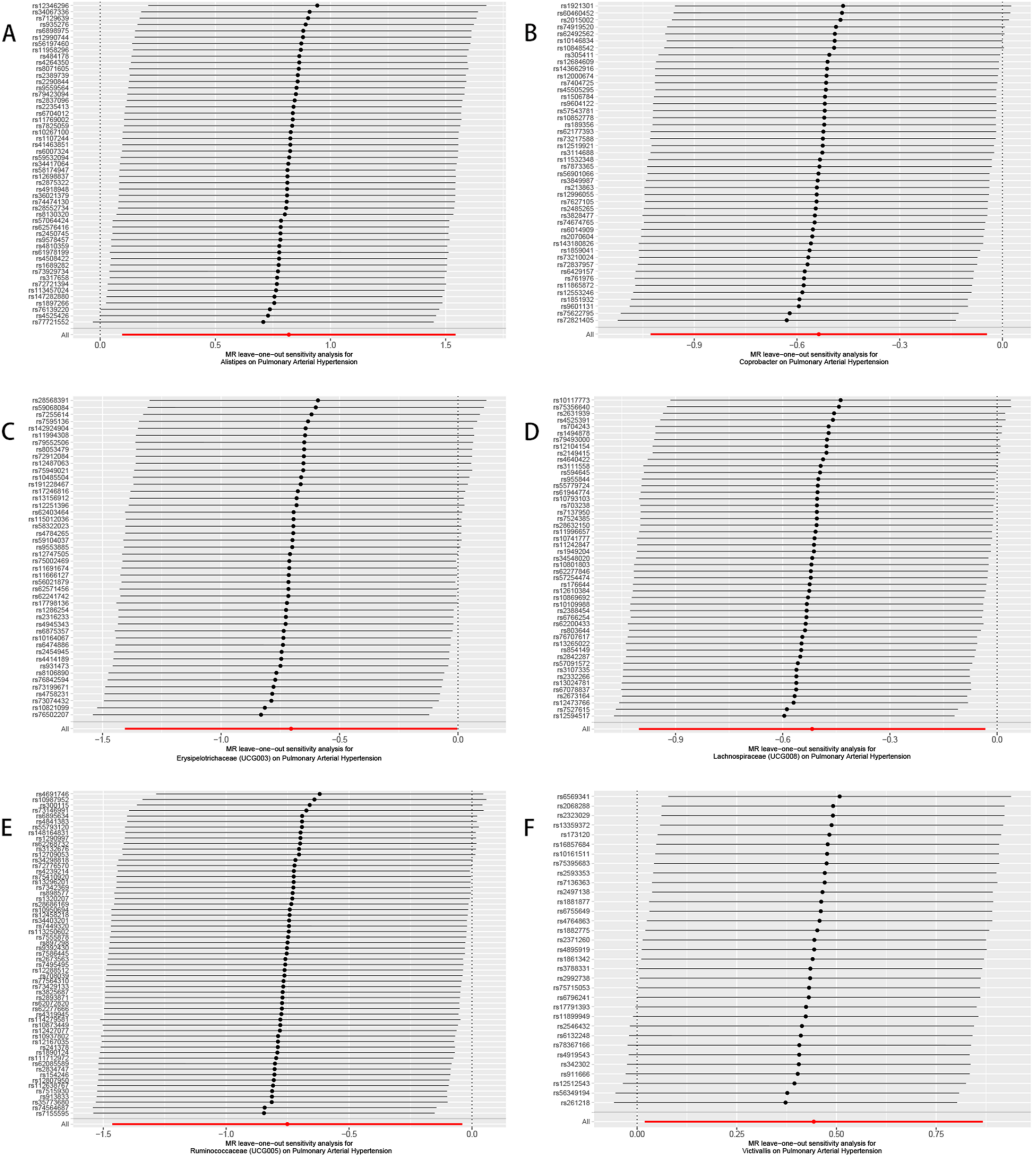
Forest plot of MR results of bacteria on pulmonary arterial hypertension. Legend: Each line represents the effect size of the corresponding SNP with the accompanying standard error. The red lines represent the average effect size of all SNP calculated by the MR egger and inverse variance weighted method respectively. MR, Mendelian randomization; SNP, single nucleotide polymorphisms

**Fig. 5:**
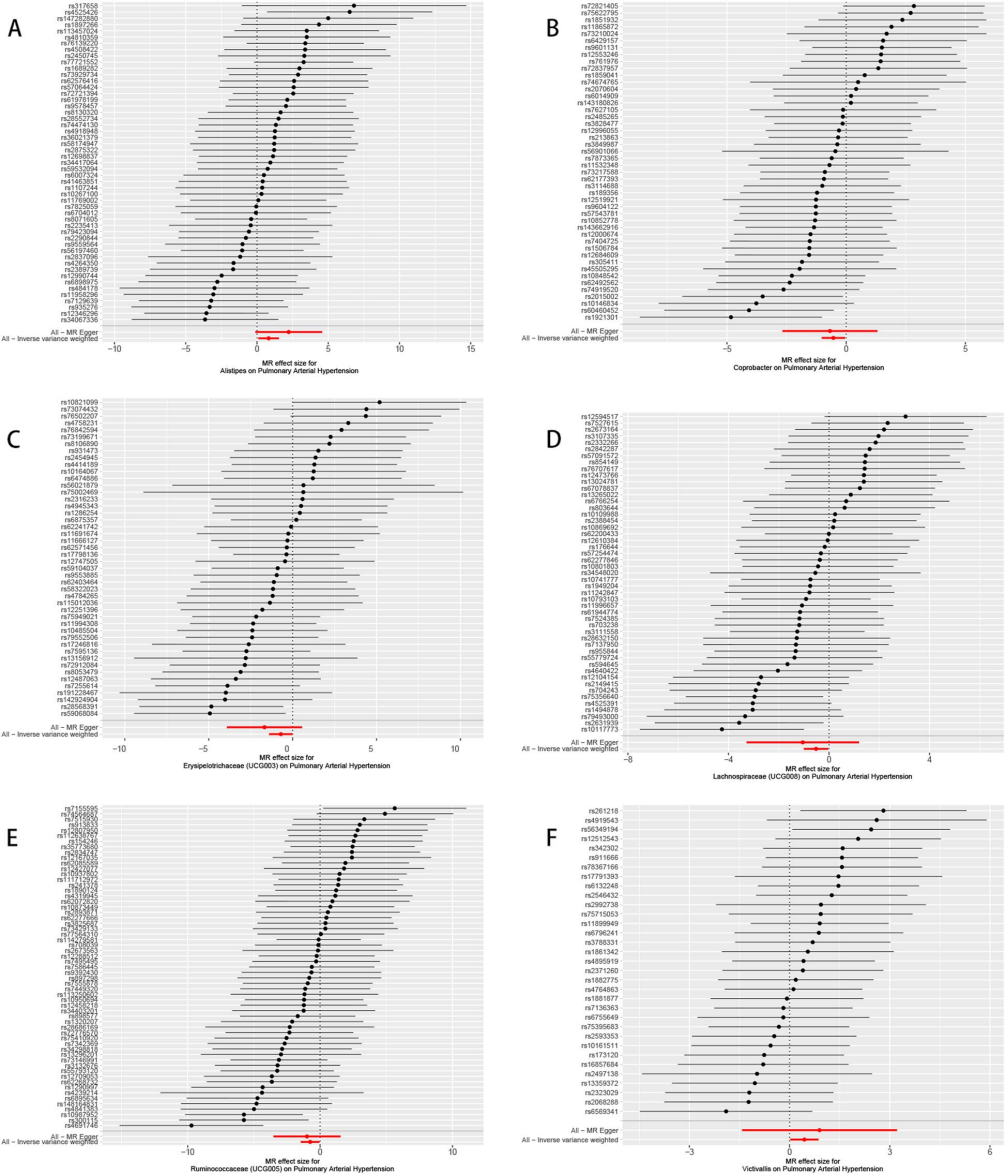
The leave-one-out analysis of MR results of bacteria on pulmonary arterial hypertension. Legend: The leave-one-out analysis removes a single SNP each time and calculates the meta-analysis effect of the remaining SNPs to observe whether the results change after removing each SNP, and if a SNP is removed, the results change greatly, indicating that the presence of the SNP has a great impact on the results. In this study, after removing a single SNP each time, the overall error bars did not change much, indicating that the results were reliable and not affected by heterogeneity. MR, Mendelian randomization; SNP, single nucleotide polymorphisms

According to the Mendelian randomisation analysis results, genetically predicted *Alistipes* (OR [odds ratio]= 2.269, 95% CI [confidence interval] 1.100–4.679, *P* = 0.027) and *Victivallis* (OR = 1.558, 95% CI 1.019–2.380, *P* = 0.040) were associated with an increased risk of PAH, while *Coprobacter* (OR = 0.585, 95% CI 0.358–0.956, *P* = 0.032), *Erysipelotrichaceae (UCG003)* (OR = 0.494, 95% CI 0.245–0.996, *P* = 0.049), *Lachnospiraceae (UCG008)* (OR = 0.596, 95% CI 0.367–0.968, *P* = 0.036), and *Ruminococcaceae (UCG005)* (OR = 0.472, 95% CI 0.231–0.962, *P* = 0.039) could causally protect against PAH (Table 1, Supplementary Table S3, Figs. 3-4). The leave-one-out plots did not suggest that any of the IVW estimates were significantly affected by individual outlier SNPs **(**Fig. 5**)**. Funnel plots demonstrated no asymmetry in the SNP estimates throughout the range of precision (Supplementary Fig. S1). Sensitivity analysis using the weighted median, weighted mode, simple mode, MR-PRESSO, and MR-Egger regression methods yielded similar results (Table 1**,** Supplementary Table S3). Cochrane’s IVW Q test showed no heterogeneity in these instrumental variables (all *P* > 0.05**,** Table 2 and Supplementary Table S4). The MR-Egger regression intercept demonstrated no significant directional pleiotropy, as all 95% CIs of the intercepts included zero (all *P* > 0.05) (Table 2 and Supplementary Table S5). The MR-PRESSO analysis did not identify any potential outlier SNPs in these bacteria, indicating no horizontal pleiotropy in the causal relationship between these bacteria and PAH (Supplementary Table S6).

**Table 2:** The heterogeneity and horizontal pleiotropy for the causal effect of gut microbiota, its metabolites and dietary patterns on pulmonary arterial hypertension

Exposure	Outcome	Method	Q	Q Df	Q *P* Value	Egger Intercept	SE	*P* value
Alistipes	PAH	IVW	41.871	50	0.786			
Alistipes	PAH	MR Egger	40.345	49	0.806	−0.090	0.073	0.223
Coprobacter	PAH	IVW	45.768	45	0.440			
Coprobacter	PAH	MR Egger	45.746	44	0.399	0.014	0.099	0.885
Erysipelotrichaceae (UCG003)	PAH	IVW	44.174	45	0.507			
Erysipelotrichaceae (UCG003)	PAH	MR Egger	43.372	44	0.498	0.073	0.082	0.376
Lachnospiraceae (UCG008)	PAH	IVW	50.782	48	0.365			
Lachnospiraceae (UCG008)	PAH	MR Egger	50.540	47	0.335	0.050	0.106	0.638
Ruminococcaceae (UCG005)	PAH	IVW	68.866	58	0.156			
Ruminococcaceae (UCG005)	PAH	MR Egger	68.822	57	0.136	0.016	0.085	0.849
Victivallis	PAH	IVW	27.383	32	0.700			
Victivallis	PAH	MR Egger	27.232	31	0.660	−0.059	0.151	0.700
Choline	PAH	IVW	59.742	94	0.998			
Choline	PAH	MR Egger	58.496	93	0.998	0.046	0.041	0.267
Carnitine	PAH	IVW	267.071	277	0.655			
Carnitine	PAH	MR Egger	267.020	276	0.640	−0.005	0.022	0.823
Betaine	PAH	IVW	58.317	58	0.464			
Betaine	PAH	MR Egger	56.251	57	0.503	0.047	0.032	0.156
TMAO	PAH	IVW	49.411	57	0.752			
TMAO	PAH	MR Egger	49.315	56	0.724	0.009	0.030	0.759
Acetate	PAH	IVW	129.309	138	0.689			
Acetate	PAH	MR Egger	129.305	137	0.668	0.002	0.035	0.952
Indolepropionate	PAH	IVW	38.354	46	0.781			
Indolepropionate	PAH	MR Egger	37.991	45	0.761	−0.031	0.052	0.550
3-Hydroxybutyrate	PAH	IVW	104.914	124	0.892			
3-Hydroxybutyrate	PAH	MR Egger	103.351	123	0.900	0.044	0.035	0.214

*Df* degree of freedom, *MR* Mendelian randomization, *TMAO* trimethylamine N-oxide, *PAH* pulmonary arterial hypertension, *IVW* Inverse variance weighted, *SE* standard error

### Causal effects of gut microbiota-derived metabolites and dietary parameters on PAH

Genetically predicted TMAO and its precursors, including betaine and carnitine, were not causally associated with PAH (all *P* > 0.05). Sensitivity analysis demonstrated consistent results (Table 1 and Supplementary Table S2). However, IVW demonstrated that elevated choline was suggestively correlated with an increased risk of PAH. Given the inconsistent outcomes of the sensitivity analysis, this association was considered insignificant. Both the MR-Egger regression and the IVW method demonstrated no heterogeneity in the metabolite SNPs (Table 2 and Supplementary Table S3). The MR-Egger regression tests and MR-PRESSO demonstrated that the results were not affected by pleiotropy (Table 2 and Supplementary Table S5 and Table S6).

IVW and sensitivity analyses indicated that SCFAs, including acetate, indolepropionate, and 3-hydroxybutyrate, were not associated with an increased risk of PAH (all *P* > 0.05**,** Table 1 and Supplementary Table S2). The results were not influenced by heterogeneity or pleiotropy (Supplementary Table S3–S5).

Genetically predicted dietary patterns, including alcohol use, fresh fruit intake, coffee intake, beef intake, and oily fish intake, were not causally associated with PAH (Supplementary Table S1–6). Therefore, dietary patterns did not mediate the association between gut microbiota and PAH (Supplementary Table S7).

### Causal effects of PAH on gut microbiota and associated metabolites

Sixty SNPs with an F-statistic of > 10 were selected as instrumental variables for PAH, as detailed in Supplementary Table S8. Reverse Mendelian randomisation analysis demonstrated that PAH had no causal effect on gut microbiota-derived metabolites. However, PAH contributed to increased levels of *Butyricicoccus* and *Holdemania* and decreased levels of *Clostridium (innocuum group), Defluviitaleaceae (UCG011), Eisenbergiella,* and *Ruminiclostridium 5* **(**Table 3 and Supplementary Table S9). No significant heterogeneity was observed in the selected instrumental variables, given that all the *P* values of the Cochrane IVW Q statistic were over 0.05 (Table 4 and Supplementary Table S10). The intercepts of the MR-Egger regression (Table 4 and Supplementary Table S11) and MR-PRESSO (Supplementary Table S12) analyses showed no significant horizontal pleiotropy among the causal effects of PAH on these bacteria.

**Table 3:** Causal effect of pulmonary arterial hypertension on gut microbiota and dependent metabolite

Exposure	Outcome	Method	Num of SNP	OR	95% CI	*P* value
PAH	Clostridium (innocuum group)	IVW	29	0.984	0.970–0.998	0.030
PAH	Butyricicoccus	IVW	30	1.008	1.001–1.015	0.026
PAH	Defluviitaleaceae (UCG011)	IVW	30	0.988	0.978–0.998	0.020
PAH	Eisenbergiella	IVW	29	0.985	0.973–0.997	0.018
PAH	Holdemania	IVW	30	1.010	1.000–1.020	0.048
PAH	Ruminiclostridium 5	IVW	30	0.992	0.985–0.999	0.032
PAH	Choline	IVW	57	1.000	0.994–1.005	0.891
PAH	Carnitine	IVW	24	1.000	0.999–1.001	0.916
PAH	Betaine	IVW	24	1.002	1.000–1.003	0.051
PAH	Acetate	IVW	59	1.000	0.999–1.001	0.864
PAH	Indolepropionate	IVW	24	1.001	0.998–1.004	0.413
PAH	3-Hydroxybutyrate	IVW	59	1.000	0.999–1.001	0.874

*SNP* single nucleotide polymorphism, *OR* odds ratio, *CI* confidence interval, *PAH* pulmonary arterial hypertension, *IVW* Inverse variance weighted. For the results of sensitivity analysis, please kindly refer to Supplementary Table S7 & Supplementary Table S11

**Table 4:** The heterogeneity and horizontal pleiotropy for the causal effect of pulmonary arterial hypertension on gut microbiota and its metabolites

Exposure	Outcome	Method	Q	Q Df	Q *P* Value	Egger Intercept	SE	*P* value
Clostridium (innocuum group)	PAH	IVW	28.279	28	0.450			
Clostridium (innocuum group)	PAH	MR Egger	28.279	27	0.397	−0.0003	0.025	0.991
Butyricicoccus	PAH	IVW	25.262	29	0.665			
Butyricicoccus	PAH	MR Egger	25.226	28	0.615	−0.002	0.012	0.851
Defluviitaleaceae (UCG011)	PAH	IVW	22.572	29	0.796			
Defluviitaleaceae (UCG011)	PAH	MR Egger	22.504	28	0.757	−0.004	0.017	0.797
Eisenbergiella	PAH	IVW	20.106	28	0.861			
Eisenbergiella	PAH	MR Egger	20.044	27	0.829	0.005	0.021	0.807
Holdemania	PAH	IVW	23.798	29	0.739			
Holdemania	PAH	MR Egger	23.785	28	0.693	−0.002	0.016	0.910
Ruminiclostridium5	PAH	IVW	14.705	29	0.987			
Ruminiclostridium5	PAH	MR Egger	14.604	28	0.982	0.004	0.012	0.753
Choline	PAH	IVW	49.665	56	0.712			
Choline	PAH	MR Egger	48.956	55	0.704	−0.004	0.005	0.404
Carnitine	PAH	IVW	32.506	23	0.090			
Carnitine	PAH	MR Egger	29.425	22	0.133	−0.002	0.001	0.143
Betaine	PAH	IVW	19.327	23	0.682			
Betaine	PAH	MR Egger	19.301	22	0.627	−0.0003	0.002	0.875
Acetate	PAH	IVW	68.927	58	0.154			
Acetate	PAH	MR Egger	68.733	57	0.137	0.0004	0.001	0.690
Indolepropionate	PAH	IVW	18.611	23	0.724			
Indolepropionate	PAH	MR Egger	18.376	22	0.684	0.001	0.003	0.632
3-Hydroxybutyrate	PAH	IVW	58.346	58	0.463			
3-Hydroxybutyrate	PAH	MR Egger	58.341	57	0.426	−0.0001	0.001	0.943

*Df* degree of freedom, *PAH* pulmonary arterial hypertension, *IVW* Inverse variance weighted, *SE* standard error, Since none of the genetic variants of trimethylamine N-oxide were found in the variant selection process, it was excluded from analysis

## Discussion

The current study revealed the intricate interactions between gut microbiota and PAH, contributing to the burgeoning evidence of the influence of the microbiome on cardiovascular pathologies. Our study underscores the potential protective role of specific bacterial genera against PAH, including *Coprobacter, Erysipelotrichaceae (UCG003), Lachnospiraceae (UCG008),* and *Ruminococcaceae (UCG005)*. In contrast, genera such as *Alistipes* and *Victivallis* emerged as potential risk factors, suggesting their contributory roles in PAH pathogenesis. Notably, our findings did not indicate significant mediation by dietary patterns in the microbiota–PAH axis, nor did we observe a causal association between gut microbiota-dependent metabolites and PAH.

The pivotal function of the gut microbiota in maintaining host homeostasis is well documented, with roles in nutrient metabolism, vitamin and hormone synthesis, immune system shaping, and resistance to pathogen colonisation [17]. Dysbiosis has been implicated in various conditions, including diabetes, coronary artery disease, heart failure, and hypertension [18]. Moreover, perturbations in the gut microbial composition have been documented in PH, which is characterised by reduced levels of beneficial bacteria [5], suggesting a possible association with PAH.

Studies have revealed that *Lachnospiraceae* and *Ruminococcaceae*, which are predominant in healthy individuals, are integral to gut integrity and metabolic processes [19]. Their associations with cardiovascular health indicators, such as arterial stiffness [20] and heart rate variability [21] further suggest that these bacteria contribute to cardiovascular homeostasis. A decline in the abundance of these genera in coronary artery disease [22], chronic heart failure [23], and HIV [24] highlights their beneficial roles.

Concurrently, an increase in *Alistipes* is associated with a higher risk of PAH. *Alistipes*, a potential pathogen, is associated with colorectal cancer [25], depression [25], diabetic nephropathy [26], and inflammation [25]. Its influence on hypertension pathogenesis is suggested by its positive correlation with systolic blood pressure in hypertensive individuals [27]. Similarly, the involvement of *Victivallis*, a strictly anaerobic bacterium, in the risk of PAH points to metabolic interplay, although the literature on its role is scant, warranting further investigation.

This study explored the controversial relationships between TMAO, its precursors, and PAH. Although some studies have indicated a correlation between elevated TMAO levels and adverse cardiovascular outcomes [28] [29], our MR analysis did not confirm a causal association. This is consistent with other MR studies that have questioned the causality of TMAO in cardiometabolic diseases [30, 31]. The inconclusive nature of the role of TMAO in PAH, coupled with the mixed prognostic implications [32, 33], indicates that observational associations may be confounded, highlighting the need for more detailed investigations.

However, the role of SCFAs in the development of PAH remains unclear. Despite their well-recognised systemic effects [34, 35], our findings do not support a direct causal relationship with PAH, pointing to a gap that future research should address.

The methodological strength of this study lies in the systematic evaluation of causal relationships using MR analysis, which is supported by extensive summary-level microbiota data. This approach mitigated reverse causation and confounding biases. Additionally, we investigated potential dietary confounders and validated our findings using multiple MR methods to ensure robustness.

However, this study had some limitations. Firstly, we selected SNPs for metabolites and bacteria at a suggestive locus-wide significance threshold of *P* < 5 × 10^−5^ [36], and our results might have been influenced by weak instrument bias. We performed a sensitivity analysis at the study-wide significance level of *P* < 5 × 10^−8^ and found that no SNP remained for metabolites and bacteria. Secondly, in the analysis of microbiota influences on PAH, MR only examines the impact of genetic factors that predispose individuals to specific abundances of particular microbial genera and how these genetic components influence the development of PAH. However, the impact of the environment and acquired risk factors on the causal association between bacteria and PAH could not be evaluated, which might contribute to the development of PAH. Thirdly, PAH may exhibit an association with specific microbial ‘types’ rather than taxonomic genera, and alpha diversity may also connect with PAH [37, 38]. Unfortunately, owing to the limitations of the MR method employed in our study, we were unable to evaluate the association between permatypes or enterotypes and the alpha diversity of microbiomes with PAH. We aim to investigate these issues in future studies. Fourthly, different ethnicities with PAH may have hereditary discrepancies. The current Mendelian randomisation analysis is primarily based on the European population, which limits the generalisability of the findings to other ethnic populations. However, limiting the European population may reduce the bias introduced by population stratification. Fifthly, this study explored the association between PAH and gut microbiota at the genus level. However, different species in the same genus may have discrepancies in their effects on metabolism and health, varying in the host and exposed environment. Some species may be protective, whereas others may exert detrimental effects on health. Studies at the species level can shed light on the precise causal associations between bacteria and diseases, along with the underlying pathological mechanisms. However, in the MiBioGen database, the lowest available level of bacteria was the genus level due to the limitations of 16S sequencing. Whole-genome shotgun metagenomics can address this limitation and provide insights into species-level resolution, which should be considered in future studies.

## Conclusion

Genetically predicted gut microbiota has a suggestive causal effect on PAH, and the underlying mechanism may be attributed to alternative mechanisms rather than the production of SCFAs and TMAO.

### Supplementary Information

**Supplementary Material 1.**

**Supplementary Material 2.**

**Supplementary Material 3.**

## Data Availability

All data are publicly available. Detailed information for these datasets is summarized in Table S 1 .

## Abbreviations

SNP: Single-nucleotide polymorphism
OR: Odds ratio
CI: Confidence interval
TMAO: Trimethylamine N-oxide
PAH: Pulmonary arterial hypertension
IVW: Inverse variance weighted
SCFA: Short-chain fatty acid

## References

[1] Humbert M, Kovacs G, Hoeper MM, Badagliacca R, Berger RMF, Brida M (2022). 2022 ESC/ERS guidelines for the diagnosis and treatment of pulmonary hypertension. Eur Heart J.

[2] Yang Y, Lin F, Xiao Z, Sun B, Wei Z, Liu B (2020). Investigational pharmacotherapy and immunotherapy of pulmonary arterial hypertension: an update. Biomed Pharmacother.

[3] Huang Y, Lin F, Tang R, Bao C, Zhou Q, Ye K (2022). Gut microbial metabolite trimethylamine N-oxide aggravates pulmonary hypertension. Am J Respir Cell Mol Biol.

[4] Chen YH, Yuan W, Meng LK, Zhong JC, Liu XY. The role and mechanism of gut microbiota in pulmonary arterial hypertension. Nutrients. 2022;14(20). 10.3390/nu14204278 PMC9610674 36296961

[5] Kim S, Rigatto K, Gazzana MB, Knorst MM, Richards EM, Pepine CJ (2020). Altered gut microbiome profile in patients with pulmonary arterial hypertension. Hypertension..

[6] Tan J-S, Liu N-N, Guo T-T, Hu S, Hua L (2021). Genetically predicted obesity and risk of deep vein thrombosis. Thromb Res.

[7] Kurilshikov A, Medina-Gomez C, Bacigalupe R, Radjabzadeh D, Wang J, Demirkan A (2021). Large-scale association analyses identify host factors influencing human gut microbiome composition. Nat Genet.

[8] Sanna S, van Zuydam NR, Mahajan A, Kurilshikov A, Vich Vila A, Võsa U (2019). Causal relationships among the gut microbiome, short-chain fatty acids and metabolic diseases. Nat Genet.

[9] Li P, Wang H, Guo L, Gou X, Chen G, Lin D (2022). Association between gut microbiota and preeclampsia-eclampsia: a two-sample Mendelian randomization study. BMC Med.

[10] Staiger D, Stock JH (1997). Instrumental variables with weak instruments. Econometrica..

[11] Burgess S, Dudbridge F, Thompson SG (2016). Combining information on multiple instrumental variables in Mendelian randomization: comparison of allele score and summarized data methods. Stat Med.

[12] Burgess S, Butterworth A, Thompson SG (2013). Mendelian randomization analysis with multiple genetic variants using summarized data. Genet Epidemiol.

[13] Verbanck M, Chen C-Y, Neale B, Do R (2018). Detection of widespread horizontal pleiotropy in causal relationships inferred from Mendelian randomization between complex traits and diseases. Nat Genet.

[14] Burgess S (2014). Sample size and power calculations in Mendelian randomization with a single instrumental variable and a binary outcome. Int J Epidemiol.

[15] Bowden J, Davey Smith G, Burgess S (2015). Mendelian randomization with invalid instruments: effect estimation and bias detection through egger regression. Int J Epidemiol.

[16] Hemani G, Tilling K, Davey SG (2017). Orienting the causal relationship between imprecisely measured traits using GWAS summary data. PLoS Genet.

[17] Floch MH (2011). Intestinal microecology in health and wellness. J Clin Gastroenterol.

[18] Peng J, Xiao X, Hu M, Zhang X (2018). Interaction between gut microbiome and cardiovascular disease. Life Sci.

[19] Schirmer M, Garner A, Vlamakis H, Xavier RJ (2019). Microbial genes and pathways in inflammatory bowel disease. Nat Rev Microbiol.

[20] Menni C, Lin C, Cecelja M, Mangino M, Matey-Hernandez ML, Keehn L (2018). Gut microbial diversity is associated with lower arterial stiffness in women. Eur Heart J.

[21] Tsubokawa M, Nishimura M, Mikami T, Ishida M, Hisada T, Tamada Y. Association of gut Microbial Genera with heart rate variability in the general Japanese population: the Iwaki cross-sectional research study. Metabolites. 2022;12(8). 10.3390/metabo12080730 PMC9414323 36005602

[22] Liu H, Chen X, Hu X, Niu H, Tian R, Wang H (2019). Alterations in the gut microbiome and metabolism with coronary artery disease severity. Microbiome..

[23] Zhang Z, Cai B, Sun Y, Deng H, Wang H, Qiao Z (2022). Alteration of the gut microbiota and metabolite phenylacetylglutamine in patients with severe chronic heart failure. Front Cardiovasc Med.

[24] Vujkovic-Cvijin I, Sortino O, Verheij E, Sklar J, Wit FW, Kootstra NA (2020). HIV-associated gut dysbiosis is independent of sexual practice and correlates with noncommunicable diseases. Nat Commun.

[25] Parker BJ, Wearsch PA, Veloo ACM, Rodriguez-Palacios A (2020). The genus Alistipes: gut Bacteria with emerging implications to inflammation, Cancer, and mental health. Front Immunol.

[26] Chen W, Zhang M, Guo Y, Wang Z, Liu Q, Yan R (2021). The profile and function of gut microbiota in diabetic nephropathy. Diabetes Metab Syndr Obes.

[27] Kim S, Goel R, Kumar A, Qi Y, Lobaton G, Hosaka K (2018). Imbalance of gut microbiome and intestinal epithelial barrier dysfunction in patients with high blood pressure. Clin Sci (Lond).

[28] Li J, Li Y, Ivey KL, Wang DD, Wilkinson JE, Franke A (2022). Interplay between diet and gut microbiome, and circulating concentrations of trimethylamine N-oxide: findings from a longitudinal cohort of US men. Gut..

[29] Heianza Y, Ma W, Manson JE, Rexrode KM, Qi L. Gut microbiota metabolites and risk of major adverse cardiovascular disease events and death: a systematic review and Meta-analysis of prospective studies. J Am Heart Assoc. 2017;6(7). 10.1161/JAHA.116.004947 PMC5586261 28663251

[30] Jia J, Dou P, Gao M, Kong X, Li C, Liu Z (2019). Assessment of causal direction between gut microbiota-dependent metabolites and Cardiometabolic health: a bidirectional Mendelian randomization analysis. Diabetes..

[31] Gagnon E, Mitchell PL, Manikpurage HD, Abner E, Taba N, Esko T (2023). Impact of the gut microbiota and associated metabolites on cardiometabolic traits, chronic diseases and human longevity: a Mendelian randomization study. J Transl Med.

[32] Yang Y, Zeng Q, Gao J, Yang B, Zhou J, Li K (2022). High-circulating gut microbiota-dependent metabolite trimethylamine N-oxide is associated with poor prognosis in pulmonary arterial hypertension. Eur Heart J Open.

[33] Videja M, Vilskersts R, Korzh S, Cirule H, Sevostjanovs E, Dambrova M (2020). Microbiota-derived metabolite trimethylamine N-oxide protects mitochondrial energy metabolism and cardiac functionality in a rat model of right ventricle heart failure. Front Cell Dev Biol.

[34] Ratajczak W, Rył A, Mizerski A, Walczakiewicz K, Sipak O, Laszczyńska M. Immunomodulatory potential of gut microbiome-derived short-chain fatty acids (SCFAs). Acta Biochim Pol. 2019;66(1). 10.18388/abp.2018_2648 30831575

[35] Tan J, McKenzie C, Potamitis M, Thorburn AN, Mackay CR, Macia L, Alt FW (2014). Chapter three - the role of short-chain fatty acids in health and disease. Advances in immunology.

[36] Luo Q, Hu Y, Chen X, Luo Y, Chen J, Wang H (2022). Effects of gut microbiota and metabolites on heart failure and its risk factors: a two-sample Mendelian randomization study. Front Nutr.

[37] Christensen L, Roager HM, Astrup A, Hjorth MF (2018). Microbial enterotypes in personalized nutrition and obesity management. Am J Clin Nutr.

[38] Klimenko NS, Tyakht AV, Popenko AS, Vasiliev AS, Altukhov IA, Ischenko DS, et al. Microbiome responses to an uncontrolled short-term diet intervention in the frame of the citizen science project. Nutrients. 2018;10(5). 10.3390/nu10050576 PMC5986456 29738477

