# Supplementary material for: Causal impact of gut microbiota and associated metabolites on pulmonary arterial hypertension: a bidirectional Mendelian randomization study
Source: BMC Pulm Med. 2024 Apr 17;24:185. doi: 10.1186/s12890-024-03008-7 (PMC11025270; doi:10.1186/s12890-024-03008-7)

Supplementary Figure

Figure S1

**Title: Funnel plot of MR results of gut microbiota on pulmonary arterial hypertension**

Legend: Funnel plot evaluates the heterogeneity among single nucleotide polymorphisms. The point significantly deviating from the overall indicates heterogeneity.


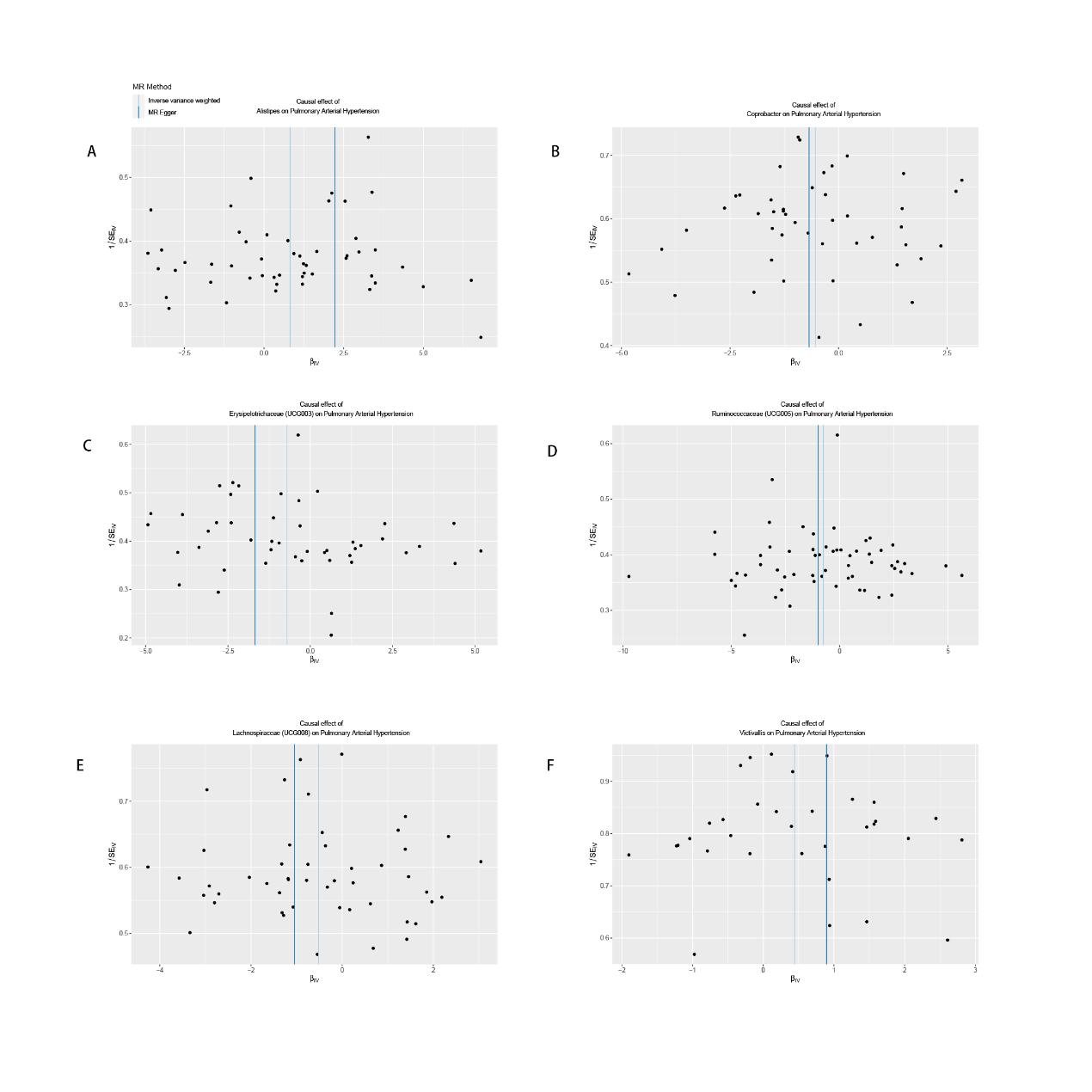

Supplement: Supplementary file 1 — Supplementary Material 1. [file 12890_2024_3008_MOESM1_ESM.docx]
